# The middle rectal artery detected by contrast-enhanced magnetic resonance imaging predicts lateral lymph node metastasis in lower rectal cancer

**DOI:** 10.1007/s00384-021-03887-w

**Published:** 2021-02-22

**Authors:** Yosuke Iwasa, Fumikazu Koyama, Nagaaki Marugami, Hiroyuki Kuge, Takayuki Nakamoto, Shinsaku Obara, Satoshi Nishiwada, Takeshi Takei, Tomomi Sadamitsu, Satoshi Yamauchi, Kimihiko Kichikawa, Masayuki Sho

**Affiliations:** 1 Department of Surgery, Nara Medical University, 840 Shijo-cho, Kashihara, 634-8522 Nara Japan; 2 Division of Endoscopy, Nara Medical University Hospital, 840 Shijo-cho, Kashihara, 634-8522 Nara Japan; 3 Department of Radiology, Nara Medical University, 840 Shijo-cho, Kashihara, 634-8522 Nara Japan

**Keywords:** Rectal cancer, Lateral lymph node metastasis, Magnetic resonance imaging, Middle rectal artery

## Abstract

**Purpose:**

Lateral lymph node (LLN) metastasis is one of the leading causes of local recurrence in patients with lower rectal cancer. Unfortunately, no diagnostic biomarkers are currently available that can predict LLN metastasis preoperatively. Accordingly, we investigated the relationship between the middle rectal artery (MRA) identified by contrast-enhanced magnetic resonance imaging (ceMRI) and LLN metastases.

**Methods:**

Data from 102 patients with lower rectal cancer who underwent surgery, and were evaluated by preoperative ceMRI, between 2008 and 2016 were reviewed retrospectively. Two expert radiologists evaluated the MRA findings. The diagnostic performance of MRA for LLN metastasis was evaluated by a multivariate analysis with conventional clinicopathological factors.

**Results:**

The MRA was detected in 67 patients (65.7%), including 32 (31.4%) with bilateral MRA and 35 (34.3%) with unilateral MRA. The tumor size, presence of the MRA, and clinical LLN status were significantly correlated with LLN metastasis. A multivariate analysis demonstrated that the presence of MRA (*P =* 0.045) and clinical LLN status (*P =* 0.001) were independent predictive factors for LLN metastasis. Furthermore, the sensitivity and negative predictive value of MRA for LLN metastasis were 95% and 97.1%, respectively.

**Conclusion:**

We successfully demonstrated that MRAs could be clearly detected by ceMRI, and the presence of MRA robustly predicted LLN metastasis in patients with lower rectal cancer, highlighting its clinical significance in the selection of more appropriate treatment strategies.

**Trial registration:**

Trial registration number: retrospectively registered 2126

Trial registration date of registration: August 23, 2019

## Introduction

Local recurrence of rectal cancer remains an important clinical problem associated with poor survival, severe morbidity, and low likelihood if salvage. Lateral lymph node (LLN) metastasis, which occurs in approximately 15% of patients, is a leading cause of local recurrence in patients with lower rectal cancer [1]. In recent years, total mesorectum excision with a clear circumferential resection margin has become an established procedure for reducing local recurrence worldwide; however, the standard treatment strategies for the LLN area, such as chemoradiotherapy and LLN dissection, differ between western countries and eastern Asian countries in cases of lower rectal cancer [2–8]. Both neoadjuvant chemoradiotherapy and LLN dissection can reduce local recurrence after surgery; however, even chemoradiotherapy induces increased complications not only perioperatively but also postoperatively, including urinary, sexual, and bowel dysfunction [9–13]. This highlights the importance of pre-treatment diagnostic tools for risk stratification of LLN metastasis, which can facilitate decision-making for reducing unnecessarily excessive treatment for LLNs in patients with lower rectal cancer.

However, the pre-treatment diagnosis of the presence of LLN metastasis remains clinically challenging. Currently, the identification of enlarged LLNs evaluated on computed tomography (CT) and/or magnetic resonance imaging (MRI) is reportedly a risk factor for LLN metastasis [14–16]; however, the clinical LLN status as diagnosed by preoperative radiological modalities has not been established.

LLN metastasis has been considered associated with lateral lymphatic drainage along the middle rectal artery (MRA) [17, 18]. Traditionally, the MRA has been described as an artery that penetrates the pelvic plexus from the lateral side along the lateral ligament. However, cadaveric anatomical studies have shown the existence of other types of MRAs and a wide range of MRA detection rates, ranging from 12 to 97% [19–25].

Recently, advances in diagnostic imaging have enabled the visualization of the detailed vascular anatomy. Bilhim indicated that the prevalence and anatomical findings of the MRA could be detected by CT angiography/digital subtraction angiography for male patients with lower urinary tract symptoms [21]. However, it is not practical to routinely perform CT angiography, as repeated exposure to radiation is required in order to capture the MRA clearly, which is thin and small.

In the present exploratory study, for the first time, we investigated the frequency of the MRA detected by contrast-enhanced MRI (ceMRI) and successfully confirmed the utility of MRI-detectable MRAs for the identification of LLN metastasis, thus highlighting the clinical significance of this biomarker for the management of patients with lower rectal cancer.

## Patients and methods

### Patients

**Fig. 1:**
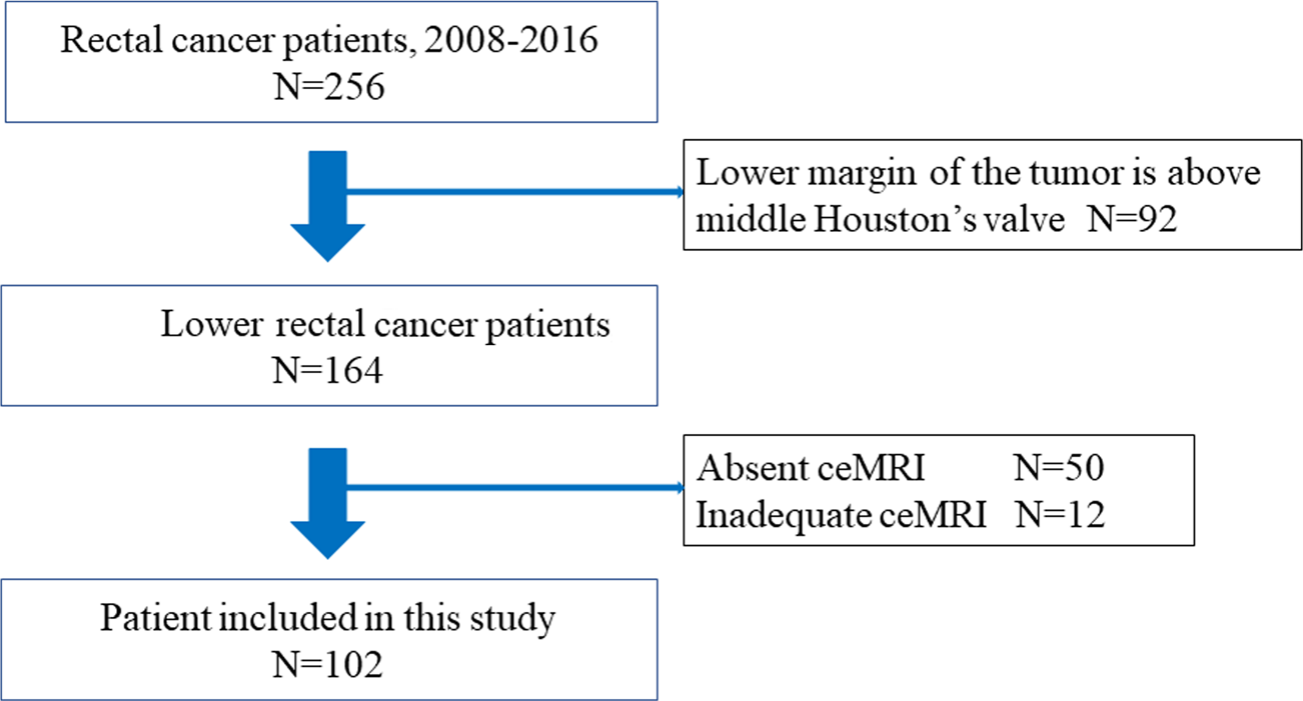
Flow diagram of this study cohort. ceMRI, contrast-enhanced magnetic resonance imaging

We retrospectively examined a total of 256 rectal cancer patients who underwent rectal resection at the Nara Medical University Hospital between January 2008 and December 2016. Lower rectal cancer was defined as a tumor with a lower end located below the middle Houston’s valve. Ninety-two patients with upper rectal cancer were excluded from this study. The exclusion criteria included absence of ceMRI (*n* = 50), insufficient contrast imaging conditions (*n* = 8), and poor images caused by body movements (*n* = 4). Ultimately, 102 patients were included in this study (Fig. 1). All lower rectal cancer patients underwent preoperative examinations including computed tomography (CT), pelvic MRI, barium enema, and colonoscopy. Bilateral LLN dissection was performed for patients diagnosed with cT3/T4 or suspected of having lymph node metastasis in the mesorectum and/or lateral pelvic area. Preservation of the autonomic nerves of the pelvic plexus was performed when LLN metastasis was not suspected. Surgically resected specimens were fixed in 10% phosphate-buffered formalin and embedded in paraffin. Tumors were classified according to the TNM staging system of the International Union Against Cancer (UICC) 8th edition. Two authorized pathologists evaluated the pathological findings.

Postoperative follow-up was performed for 5 years. Tumor markers were examined every 3 months, and all patients were evaluated using chest/abdominal contrast enhanced CT or pelvic MRI every 6 months after surgery according to the Japanese Society for Cancer of the Colon and Rectum (JSCCR) guidelines [26]. The presence of LLN metastasis was defined in the following cases: the patients who were diagnosed by a postoperative pathological examination or had LLN recurrence evaluated by imaging tests after surgery. When a new lesion was found on postoperative imaging and showed a gradual increasing trend, the lesion was diagnosed as a recurrent lesion.

This study was approved by the ethics committee of the Nara Medical University (No. 2126). All patients gave their informed consent for the use of their anonymized data via an opt-out method. Patients’ consent to participate was obtained through an opt-out method.

### Contrast-enhanced magnetic resonance imaging

We have routinely used a 1.5-T MRI scanner (MAGNETOM Avanto; SIEMENS Healthineers, Erlangen, Germany) or 3-T MRI scanner (MAGNETOM Verio, Skyra; SIEMENS Healthineers, Erlangen, Germany) for the diagnosis of the depth of the primary rectal tumor and LLN swelling. Patients underwent MRI examinations in the supine position without preparation. T1-weighted gradient-echo sequencing was performed for enhanced MRI. For contrast-enhanced MRI, Gd-DTPA, Gd-DOTA, or Gd-BT-DO3A (0.1 mmol/kg) was infused into the vein. The slice thickness of all sequences was 1 mm and with a mean total time of 30 min.

### Definition and evaluation of MRA

**Fig. 2:**
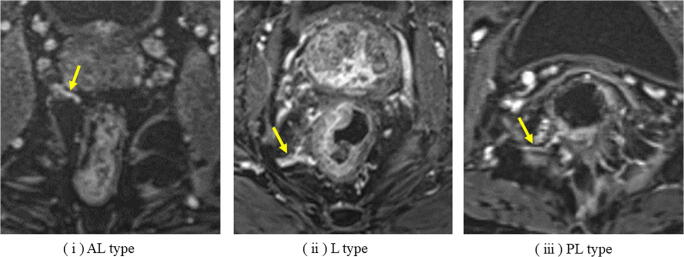
Three types of middle rectal artery detected by contrast-enhanced MRI axial slices. (i) AL type, antero-lateral type; (ii) L type, lateral type; (iii) PL type, postero-lateral type

Kiyomatsu et al. reviewed and classified MRAs into the following three branches: (i) antero-lateral (AL) type, which branches from the prostatic artery, inferior vesical artery, or uterine artery; (ii) lateral (L) type, which runs into the mesorectum via the lateral ligament; and (iii) postero-lateral (PL) type, which runs into the rectosacral fascia consisted of posterior rectal wall (Fig. 2) [27]. In the present study, the MRA was defined as present when each artery was observed as running into the rectum from the outside of the mesorectum on axial slices of ceMRI. The presence of MRA was evaluated by two expert radiologists blinded to the clinicopathological data.

### Definition of the clinical LLN status and adaptation of LLN dissection

In this study, we defined as cases as having a positive clinical LLN status when the LLN short axis exceeded 7 mm on preoperative pelvic MRI, with reference to previously reports [14, 15]. LLN dissection was adapted for lower rectal cancer patients with cT3/T4, suspected of having proximal lymph node metastasis, as well as positive clinical LLN status. We performed neoadjuvant chemotherapy (NAC) for such patients starting in 2014, and 21 patients received NAC in this study. Twenty-one patients received NAC, among whom 20 underwent LLN dissection.

### Statistical analyses

Statistical analyses were performed using the SPSS software program, version 25.0 (IBM, Armonk, NY, USA). Categorical variables were expressed as numbers and percentages. In order to evaluate the relationship between the presence of the MRA and LLN metastasis, the sensitivity, specificity, positive predictive value, and negative predictive value were calculated. Fisher’s exact test and the chi-squared test were used as appropriate to analyze the significantly different clinical factors between LLN metastasis-positive and metastasis-negative patients. Statistical analyses of all variables were considered significant at *P*<0.05. Multivariate logistic regression analysis was employed to evaluate clinicopathological variables and the presence of MRA that were significant on a univariate analysis for the detection of LLN metastasis.

## Results

### Patients’ characteristics

Patients’ clinicopathological characteristics are shown in Table 1. The 102 total patients with a median age of 64 (range 30–82) years old included 64 men and 38 women. The median postoperative follow-up period was 49.1 months. A total of 16 (15.7%) patients were diagnosed as LLN metastasis by pathological examination after surgery. Right and left LLN metastasis was observed in 13 and 7 patients, respectively. LLN recurrences after surgery were occurred in 4 (3.9%) patients. Taken together, LLN metastasis was confirmed in a total of 20 (19.6%) patients in this study.

**Table 1:** Clinicopathological characteristics of clinical cohort

	No. of patients *N* = 102
Median age, years (range)	64 (30–82)
Sex (male/female), *n* (%)	64 (62.7)/38 (37.2)
Tumor location (Ra/Rb/P), *n* (%)	18 (17.6)/82 (80.4)/2 (2.0)
Distance from anal verge, cm (range)	4.0 (0–12.0)
CEA level, ng/mL (range)	3.95 (0.8–82.3)
CA19-9 level, U/mL (range)	11.5 (1–1823)
Lateral lymph node dissection, *n* (%)	69 (67.6)
Median tumor size, mm (range)	44 (5–130)
cT status, *n* (%)	
cT1b/cT2/cT3/cT4	7 (6.9)/17 (16.7)/54 (52.9)/24 (23.5)
cN status (proximal lymph node area), *n* (%)	
cN0/cN1/cN2	30 (29.4)/52 (51.0)/20 (19.6)
Clinical lateral lymph node status, *n* (%)	
cLLN positive/cLLN negative	26 (25.5)/76 (74.5)
Distant metastasis, *n* (%)	
cM0/cM1	91 (89.2)/11 (10.8)
Neoadjuvant chemotherapy, *n* (%)	
Yes/no	21 (20.6)/81 (79.4)
Median postoperative period, months (range)	49.1 (3.8–121.9)
(y)pT status, *n* (%)	
(y)pT0/(y)pT1b/(y)pT2/(y)pT3/(y)pT4	1 (1.0)/7 (6.9)/24 (23.5)/56 (54.9)/14 (13.7)
Proximal lymph node metastasis, *n* (%)	
(y)pN0/(y)pN1/(y)pN2	43 (42.2)/29 (28.4)/30 (29.4)
Lateral lymph node metastasis, *n* (%)	16 (15.7)
Right side/left side	13 (12.7)/7 (6.9)
Lymphatic invasion, *n* (%)	
Positive/negative	84 (82.4)/18 (17.6)
Venous invasion, *n* (%)	
Positive/negative	78 (76.5)/24 (23.5)
Histopathological grade, *n* (%)	
G1/G2/G3	32 (31.4)/55 (53.9)/15 (14.7)
Tumor resection margin, *n* (%)	
R0/R1	93 (91.2)/9 (8.8)
Lateral lymph node recurrence, *n* (%)	
Present/absent	4 (3.9)/98 (96.1)

*LLN* lateral lymph node, *CEA* carcinoembryonic antigen, *CA19-9* carbohydrate antigen 19-9

### The detection rate of MRA by ceMRI

Using the ceMRI examinations, MRAs were detected in 67 (65.7%) patients, and the numbers of AL/L/PL MRAs on the right side were 28 (27.5%)/35 (34.3%)/22 (21.6%), while that on the left side were 16 (15.7%)/25 (24.5%)/18 (17.6%), respectively. There were 32 (31.4%) patients with bilateral MRAs and 35 (34.3%) with unilateral MRAs. Thirty-five (34.3%) patients had no detectable MRAs (Table 2). The number of identified MRAs was 1 vessel in 24 cases, 2 in 21, 3 in 12, 4 in 7, 5 in 2, and 6 in 1, respectively.

**Table 2:** MRA detection rate using contrast-enhanced MRI

	MRA detection rate (%) *N*=102
Right side, *n* (%)	
Antero-lateral type	28 (27.5)
Lateral type	35 (34.3)
Postero-lateral type	22 (21.6)
Left side, *n* (%)	
Antero-lateral type	16 (15.7)
Lateral type	25 (24.5)
Postero-lateral type	18 (17.6)
The number of patients who have detectable MRA, *n* (%)	
Bilateral	32 (31.4)
Unilateral	35 (34.3)
Absent	35 (34.3)
The number of pelvic-halves of MRA, *n* (%)	
Present	99 (48.5%)
Absent	105 (51.5%)

*MRA* middle rectal artery, *MRI* magnetic resonance image

### The correlation between LLN metastasis and the frequency and trajectory of MRA

The incidence of LLN metastasis in the patients with MRA was higher than in those without MRA (*P=*0.002, Table 3). Furthermore, LLN metastasis was significantly more frequent in the patients with bilateral MRAs (43.8%) than in those without any MRAs (2.9%) (*P<*0.001). Importantly, regardless of the tumor side in rectum, LLN metastasis frequently developed on the same side with MRAs (21/99, 21.2%). On the other hand, there were few instances of LLN metastasis on the without side (3/105, 2.9%).

**Table 3:** Correlation between LLN metastasis and clinical and MRA status

	LLN metastasis		Univariate analysis	Multivariate analysis		
	Negative (*n* = 82)	Positive (*n* = 20)	*P* value	Odds ratio	95% C.I.	*P* value
Clinical status						
Age (<64/≥64 years)	39/43	14/6	0.072			
Sex (male/female)	53/59	11/9	0.424			
Tumor central location (Ra/Rb, P)	15/67	3/17	0.510			
Distance from anal verge (<5/≥5 cm)	40/42	14/6	0.088			
CEA level (<5/≥5 ng/mL)	52/30	13/7	0.895			
CA19-9 level (<38/≥38 U/mL)	75/7	16/4	0.141			
Tumor size (<44/≥44 mm)	37/45	14/6	0.046	1.328	0.389–4.537	0.651
cT status (≤cT1, T2/≥cT3, T4)	21/61	3/17	0.245			
cN status (proximal lymph node area) (positive/negative)	56/26	16/4	0.303			
Clinical LLN status (positive/negative)	13/69	13/7	<0.001	7.409	2.316–23.697	0.001
Neoadjuvant chemotherapy (yes/no)	13/69	8/12	0.023			
Distant metastasis (cM0/cM1)	74/8	17/3	0.369			
Tumor resection margin (R0/R1)	75/7	18/2	0.561			
MRA status evaluated by ceMRI						
MRA (positive/negative)	48/34	19/1	0.002	8.922	1.045–76.159	0.045
Laterality of MRA			<0.001			
Bilateral/unilateral/absent	18/30/34	14/5/1				

*LLN* lateral lymph node, *MRA* middle rectal artery, *CEA* carcinoembryonic antigen, *CA19-9* carbohydrate antigen 19-9, *AL type* antero-lateral type, *L type* lateral type, *PL type* postero-lateral type

### Multivariate analysis of predictive factors associated with LLN metastasis

The correlation between the clinical factors and LLN metastasis is shown in Table 3. The tumor size, presence of MRA, and clinical LLN status were significantly correlated with LLN metastasis. A multivariate analysis revealed that the presence of MRA (*P*=0.045) and clinical lateral lymph node status (*P*=0.001) emerged as significantly independent predictive factors for LLN metastasis.

### The diagnostic accuracy of the MRAs detected by ceMRI examination for predicting LLN metastasis

In this study, the diagnostic performance of the presence of MRAs detected by ceMRI is an important predictive factor for LLN metastasis. The sensitivity, specificity, accuracy, positive predictive value, and negative predictive value of the presence of MRAs detected by MRI for LLN metastasis were 95% (19/20), 41.5% (34/82), 61.8% (63/102), 28.4% (19/67), and 97.1% (34/35), respectively (Table 4).

**Table 4:** Diagnostic accuracy of MRA detected by contrasted-enhance MRI for LLN metastasis

		LLN metastasis			PPV (%)	NPV (%)
		Positive	Negative	Total		
MRA	Positive	19	48	67	28.4	97.1
	Negative	1	34	35		

*MRA* middle rectal artery, *LLN* lateral lymph node, *PPV* positive predictive value, *NPV* negative predictive value

## Discussion

In order to advance cancer treatment into a new era of precision medicine, the development of individualized treatment strategies for cancer patients is essential. In this exploratory study, we investigated for the first time the presence of MRAs using ceMRI and identified a significant correlation between MRAs and LLN metastasis. MRAs have been investigated anatomically using cadavers in several previous studies; however, clinical and radiological studies have not been conducted. The cadaveric studies mainly discussed the frequency, origin, and/or trajectory of MRAs. These anatomical studies have shown a wide range of detection rates because of variation in the definition of MRA. Kiyomatsu et al. reviewed the frequency and trajectory of MRA in detail and described three types of MRAs [27]. We therefore adapted this classification for the definition of MRAs in the present study because it covers almost all reported running patterns of MRAs.

For the preoperative evaluation of lower rectal cancer, high-resolution T2-weighted turbo spin-echo images are generally used for assessing the depth of tumor invasion, circumferential resection margin, and lymph node metastasis [28, 29]. In addition, gadolinium-enhanced and multiparametric MRI is used for staging the T status and evaluation of the efficacy of preoperative treatment [30, 31]. Since T1-weighted gradient-echo sequences can counteract the effects of adipose tissue and calcification in the mesorectum, ceMRI reveals a satisfactory performance for the identification of MRAs. Since MRAs were clearly visualized in the arterial phase of ceMRI, we used their presence as an indicator of lymphatic flow to the LLN region. While veins can be visualized with ceMRI, they are more difficult to evaluate than arteries because they are complex and ambiguous. Therefore, we thought that it was simpler and more practical to use MRA identified by ceMRI as a potent predictor of LLN metastasis.

In the present study, the prevalence of LLN metastasis was 19.6%, which is consistent with previous reports [2, 32, 33]. From a clinical viewpoint, regarding LLN metastasis in lower rectal cancer, preoperative biomarkers for predicting LLN metastasis should have excellent negative predictive values, that is because it can facilitate to reduce unnecessary lymph node dissection and/or chemoradiotherapy. The MRA status demonstrated an adequate predictive performance for LLN metastasis in this study, with a sensitivity and negative predictive value of 95% and 97.1%, respectively. Furthermore, in cases with no MRAs on one side, the rate of LLN metastasis on the same side was extremely limited to 2.9%. These results highlight that MRA may help physicians and patients make decisions to avoid invasive treatments for LLN metastasis.

The gender, tumor location, proximal lymph node metastasis, tumor depth of invasion, lymphatic or venous invasion, histological tumor differentiation, and tumor size have been reported as risk factors for LLN metastasis in previous reports [2, 3]. Based on our multivariate analysis, the clinical LLN status and MRA identification on ceMRI were extracted as independent risk factors. In the present study, we defined LLN with a short axis of more than 7 mm as clinical LLN metastasis on preoperative pelvic MRI. The sensitivity and negative predictive value were 65.0% and 90.8%, respectively. Fujita recently reported that the LLN diameter exceeding 5 mm was a risk factor for LLN metastasis [34]. However, the sensitivity and negative predictive value in that report were limited at 62% and 89%, respectively. Fujita’s findings and our own indicate that there are a certain number of patients who have LLN metastasis without LLN swelling. Micro-metastasis without LLN swelling cannot be identified using the selection criteria based on the lymph node diameter. Coy et al. and Shimoyama reported that about 20% of patients with negative LLN metastasis had micro-metastasis detected using immunohistochemical staining [35, 36]. Our results indicate that the assessment of the presence of MRAs by ceMRI precisely predicts LLN metastasis (including micro-metastasis) on each side in patients with lower rectal cancer.

Several limitations associated with the present study warrant mention. First, this study was a single-center, retrospective study. Second, we analyzed MRAs in a moderately sized clinical cohort. Third, no all MRAs are always going to be detected by ceMRI, although most of them may be visualized on 1-mm slice pelvic images. Finally, we need to prove the reproducibility of MRAs, since this is the first study. Further prospective studies are therefore needed in order to verify the detectability of MRAs by MRI via an anatomical approach and its validity as a significant predictor of LLN metastasis.

In conclusion, we successfully showed that MRAs could be clearly detected by ceMRI, and MRAs robustly predict LLN metastasis in patients with lower rectal cancer, highlighting their clinical significance in the selection of more appropriate treatment strategies. Furthermore, our study provides a framework for developing individualized treatment strategies for LLN and designing future clinical trials for patients with lower rectal cancer.

## Data Availability

Data sharing is not applicable in this study.

## References

[1] Chen JN, Liu Z, Wang ZJ, Mei SW, Shen HY, Li J, Pei W, Wang Z, Wang XS, Yu J, Liu Q (2020). Selective lateral lymph node dissection after neoadjuvant chemoradiotherapy in rectal cancer. World J Gastroenterol.

[2] Sugihara K, Kobayashi H, Kato T, Mori T, Mochizuki H, Kameoka S, Shirouzu K, Muto T (2006). Indication and benefit of pelvic sidewall dissection for rectal cancer. Dis Colon Rectum.

[3] Akiyoshi T, Watanabe T, Miyata S, Kotake K, Muto T, Sugihara K, Japanese Society for Cancer of the Colon and Rectum (2012). Results of a Japanese nationwide multi-institutional study on lateral pelvic lymph node metastasis in low rectal cancer: Is it regional or distant disease?. Ann Surg.

[4] Fujita S, Mizusawa J, Kanemitsu Y, Ito M, Kinugasa Y, Komori K, Ohue M, Ota M, Akazai Y, Shiozawa M, Yamaguchi T, Bandou H, Katsumata K, Murata K, Akagi Y, Takiguchi N, Saida Y, Nakamura K, Fukuda H, Akasu T, Moriya Y, Colorectal Cancer Study Group of Japan Clinical Oncology Group (2017). Mesorectal excision with or without lateral lymph node dissection for clinical stage II/III lower rectal cancer (JCOG0212). Ann Surg.

[5] Cedermark B, Dahlberg M, Glimelius B, Pahlman L, Rutqvist LE, Wilking N, Swedish Rectal Cancer Trial (1997). Improved survival with preoperative radiotherapy in resectable rectal cancer. N Engl J Med.

[6] Kapiteijn E, Marijnen CA, Nagtegaal ID, Putter H, Steup WH, Wiggers T (2001). Preoperative radiotherapy combined with total mesorectal excision for resectable rectal cancer. N Engl J Med.

[7] Sauer R, Becker H, Hohenberger W, Rodel C, Wittekind C, Fietkau R (2004). Preoperative versus postoperative chemoradiotherapy for rectal cancer. N Engl J Med.

[8] Kim MJ, Kim TH, Kim DY, Kim SY, Baek JY, Chang HJ, Park SC, Park JW, Oh JH (2015). Can chemoradiation allow for omission of lateral pelvic node dissection for locally advanced rectal cancer?. J Surg Oncol.

[9] Marijnen CA, van de Velde CJ, Putter H, van den Brink M, Maas CP, Martijn H (2005). Impact of short-term preoperative radiotherapy on health-related quality of life and sexual functioning in primary rectal cancer: report of a multicenter randomized trial. J Clin Oncol.

[10] Peeters KC, van de Velde CJ, Leer JW, Martijn H, Junggeburt JM, Kranenbarg EK (2005). Late side effects of short-course preoperative radiotherapy combined with total mesorectal excision for rectal cancer: increased bowel dysfunction in irradiated patients―a Dutch colorectal cancer group study. J Clin Oncol.

[11] Swellengrebel HA, Marijnen CA, Verwaal VJ, Vincent A, Heuff G, Gerhards MF (2011). Toxicity and complications of preoperative chemoradiotherapy for locally advanced rectal cancer. Br J Surg.

[12] Fujita S, Akasu T, Mizusawa J, Saito N, Kinugasa Y, Kanemitsu Y, Ohue M, Fujii S, Shiozawa M, Yamaguchi T, Moriya Y, Colorectal Cancer Study Group of Japan Clinical Oncology Group (2012). Colorectal Cancer Study Group of Japan Clinical Oncology Group: Postoperative morbidity and mortality after mesorectal excision with and without lateral lymph node dissection for clinical stage II or stage III lower rectal cancer (JCOG 0212): results from a multicenter, randomized controlled, non-inferiority trial. Lancet Oncol.

[13] Saito S, Fujita S, Mizusawa J, Kanemitsu Y, Saito N, Kinugasa Y, Akazai Y, Ota M, Ohue M, Komori K, Shiozawa M, Yamaguchi T, Akasu T, Moriya Y, Colorectal Cancer Study Group of Japan Clinical Oncology Group (2016). Colorectal Cancer Study Group of Japan Clinical Oncology Group: Male sexual dysfunction after rectal cancer surgery: results of a randomized trial comparing mesorectal excision with and without lateral lymph node dissection for patients with lower rectal cancer: Japan Clinical Oncology Group Study JCOG0212. Eur J Surg Oncol.

[14] Yano H, Saito Y, Takeshita E, Miyake O, Ishizuka N (2007). Prediction of lateral pelvic node involvement in low rectal cancer by conventional computed tomography. Br J Surg.

[15] Arii K, Takifuji K, Yokoyama S, Matsuda K, Higashiguchi T, Yamaue H (2006). Preoperative evaluation of pelvic lateral lymph node of patients with lower rectal cancer: comparison study of MR imaging and CT in 53 patients. Langenbeck's Arch Surg.

[16] Ogura A, Konishi T, Cunningham C, Garcia-Aguilar J, Iversen H, Toda S, Lee IK, Lee HX, Uehara K, Lee P, Putter H, van de Velde CJH, Beets GL, Rutten HJT, Kusters M, on behalf of the Lateral Node Study Consortium (2019). Neoadjuvant (chemo)radiotherapy with total mesorectal excision only is not sufficient to prevent lateral local recurrence in enlarged nodes: results of the multicenter lateral node study of patients with low cT3/4 rectal cancer. J Clin Oncol.

[17] Blair JB, Holyoke EA, Best RR (1950). A note on the lymphatics of the middle and lower rectum and anus. Anat Rec.

[18] Miscusi G, Masoni L, Dell’Anna A, Montori A (1987). Normal lymphatic drainage of the rectum and the anal canal revealed by lymphoscintigraphy. Colo-proctology.

[19] Sato K, Sato T (1991). The vascular and neuronal composition of the lateral ligament of the rectum and the rectosacral fascia. Surg Radiol Anat.

[20] Didio LJ, Diaz-Franco C, Schemainda R, Bezerra AJ (1986). Morphology of the middle rectal arteries. A study of 30 cadaveric dissections. Surg Radiol Anat.

[21] Bilhim T, Pereira JA, Tinto HR, Fernandes L, Duarte M, O’Neill JE, Pisco JM (2013). Middle rectal artery: myth or reality? Retrospective study with CT angiography and digital subtraction angiography. Surg Radiol Anat.

[22] Jones OM, Smeulders N, Wiseman O, Miller R (1999). Lateral ligaments of the rectum: an anatomical study. Br J Surg.

[23] Nano M, Dal Corso HM, Lanfranco G, Ferronato M, Hornung JP (2000). Contribution to the surgical anatomy of the ligaments of the rectum. Dis Colon Rectum.

[24] Boxall TA, Smart PJ, Griffiths JD (1963). The blood-supply of the distal segment of the rectum in anterior resection. Br J Surg.

[25] Ayoub SF (1978). Arterial supply to the human rectum. Acta Anat.

[26] Hashiguchi Y, Muro K, Saito Y, Ito Y, Ajioka Y, Sugihara K (2020). Japanese Society for Cancer of the Colon and Rectum (JSCCR) guidelines 2019 for the treatment of colorectal cancer. Int J Clin Oncol.

[27] Kiyomatsu T, Ishihara S, Murono K, Otani K, Yasuda K, Nishikawa T, Tanaka T, Hata K, Kawai K, Nozawa H, Yamaguchi H, Watanabe T (2017). Anatomy of the middle rectal artery: a review of the historical literature. Surg Today.

[28] Nougaret S, Reinhold C, Mikhael HW, Rouanet P, Bibeau F, Brown G (2013). The use of MR imaging in treatment planning for patients with rectal carcinoma: have you checked the “DISTANCE”?. Radiology.

[29] Al-Sukhni E, Milot L, Fruitman M, Beyene J, Victor JC, Schmocker S (2012). Diagnostic accuracy of MRI for assessment of T category, lymph node metastases, and circumferential resection margin involvement in patients with rectal cancer: a systematic review and meta-analysis. Ann Surg Oncol.

[30] Hötker AM, Garcia-Aguilar J, Gollub MJ (2014). Multiparametric MRI of rectal cancer in the assessment of response to therapy: a systematic review. Dis Colon Rectum.

[31] Tamakawa M, Kawaai Y, Shirase R, Satoh T, Akiba H, Hyodoh H, Hareyama M, Furuhata T, Hirata K, Hasegawa T (2010). Gadolinium-enhanced dynamic magnetic resonance imaging with endorectal coil for local staging of rectal cancer. Jpn J Radiol.

[32] Fujita S, Yamamoto S, Akasu T, Moriya Y (2003). Lateral pelvic lymph node dissection for advanced lower rectal cancer. Br J Surg.

[33] Ueno M, Oya M, Azekura K, Yamaguchi T, Muto T (2005). Incidence and prognostic significance of lateral lymph node metastasis in patients with advanced low rectal cancer. Br J Surg.

[34] Fujita S, Yamamoto S, Akasu T, Moriya Y (2009). Risk factors of lateral pelvic lymph node metastasis in advanced rectal cancer. Int J Color Dis.

[35] Coy CS, Meirelles LR, Leal RF, Ayrizono ML, Goes JR, Fagundes JJ (2010). Evaluation of lateral lymph node metastasis in advanced distal rectal cancer. Hepatogastroenterology.

[36] Shimoyama M, Yamazaki T, Suda T, Hatakeyama K (2003). Prognostic significance of lateral lymph node micrometastases in low rectal cancer: an immunohistochemical study with CAM5.2. Dis Colon Rectum.

